# Urban Seismic Risk Assessment by Integrating Direct Economic Loss and Loss of Statistical Life: An Empirical Study in Xiamen, China

**DOI:** 10.3390/ijerph17218154

**Published:** 2020-11-04

**Authors:** Shutian Zhou, Guofang Zhai, Yijun Shi, Yuwen Lu

**Affiliations:** 1 School of Arts (School of Architecture), Nantong University, Nantong 226001, China; 2 School of Architecture and Urban Planning, Nanjing University, Nanjing 210093, China; guofang_zhai@nju.edu.cn (G.Z.); yuwen_lu@smail.nju.edu.cn (Y.L.); 3 School of Landscape Architecture, Zhejiang A&F University, Hangzhou 311300, China; yijun_shi@zafu.edu.cn

**Keywords:** seismic risk, disaster losses, risk assessment approach, quantitative methods, value of statistical life, direct economic loss

## Abstract

The growing densities of human and economic activities in cities lead to more severe consequences when a catastrophe such as an earthquake occurs. This study on urban seismic risk evaluation is carried out from the perspective of the direct loss caused by disasters in urban areas, including the measurement of both the expected direct economic loss and loss of life in the face of characteristic earthquakes. Aiming to estimate, quantify and visualize the earthquake risk in each unit of urban space, this research proposes to assess urban seismic risk by integrating the direct economic loss and the loss of statistical life in a disaster, with consideration of diverse earthquake frequencies. Empirical research of the proposed assessment framework and corresponding models is then conducted to measure urban seismic risk in Xiamen, China. Key findings of the case study include the expected direct economic losses and the expected number of deaths in three characteristic earthquakes, their estimated spatial distributions, the average loss of the value of a statistical life (VSL) of one average local resident and the overall seismic risk distributions in Xiamen.

## 1. Introduction

Earthquakes are a serious natural hazard in many parts of the world and are often associated with severe property damage and loss of life [1]. As the most complicated social ecological system and the most important carrier of human activities, cities often show great fragility in the face of all kinds of natural disasters, such as earthquakes, storms, mountain fires, etc., leading to high casualties and economic losses, which is increasingly a problem, especially in developing countries. China is urbanizing at a rapid pace and facing deepening contradictions between population, resources and the environment, making the frequent occurrence of disasters one of the most vital challenges to sustainable development. As human beings enter the age of a risk society, governments and academia have attached increasing importance to natural disaster risk analysis and public safety issues, while the economic evaluation of disaster losses has become a practical problem [2].

The degree of negative consequences resulting from disasters to human society is closely related to socioeconomic development, local population density and the extent of the disaster. With capital, population and infrastructures highly concentrated in cities, urban seismic risk is growing worldwide. Ex-ante assessments of disaster losses can help us to determine the possible scope of disaster and boost the consideration of the real implications of potential natural disasters on urban socioeconomic development. They may also help to confirm the key actions required for disaster prevention and mitigation planning and the implementation of efficient construction work.

Existing earthquake records and literature on seismic disaster losses have shown that the greater the intensity of the earthquake in urban areas, the greater the damage to construction [3,4], while the destruction of buildings was usually the major and direct cause of death and injury [5,6]. Earthquake loss assessment has thus become an important basis for the government and social organizations to carry out earthquake relief works. However, when evaluating seismic risks or losses, few scholars have considered the earthquake casualties and economic losses simultaneously in a single measurement. In Chinese literature on earthquake damage estimation, more attention has been paid to building vulnerability analysis and direct economic losses due to building destructions [5,6,7,8,9]. The seismic risk assessment approach used in HAZUS (Hazards U.S, developed by the Federal Emergency Management Agency, FEMA) [6,10] and the earthquake loss studies in Europe [11] and the Euro-Mediterranean Region [12] only attempt to estimate the potential casualties caused by earthquake instead of sufficiently considering the value of losses of life in disasters. Life is priceless, and so the value of life lost in disasters should be taken very seriously. The understanding of the value of a statistical life (VSL) started as early as in the 1960s in economic academia [13,14]; however, in evaluations of diverse disaster losses and disaster risk assessment studies, there is still a certain gap in considering the loss in VSL as an important part of disaster consequences due to technical or ethical reasons [15]. How may one combine economic losses and non-economic losses into a unified dimension for quantitative evaluation in seismic risk research? In this work, we also explore how to comprehensively consider the diverse consequences of urban disasters and their different occurrence probabilities in a single measurement. There is still limited theory to answer these questions completely and systematically. 

This study on urban seismic risk evaluation, which aims to estimate, quantify and visualize the earthquake risk in each unit of urban space, is carried out from the perspective of direct loss caused by disasters in urban areas, including the measurement of both the expected direct economic loss and loss of life as a result of characteristic earthquakes. In this research, it was hypothesized that (1) the risk of a certain seismic intensity degree can be calculated through the measurement of the annual expected value of direct loss within an assessment framework, which considers diverse earthquake frequencies and integrates both the direct economic loss and the loss of VSL in a disaster; and that (2) the overall seismic risk distribution pattern is strongly affected by the building fragility in an earthquake and the distributions of construction and population in the city. 

This paper offers two main contributions. First, it provides a new perspective on how to understand urban disaster risk while considering both occurrence frequencies and consequences by redefining and measuring the “annual expected direct loss”. On this basis, we propose an urban seismic risk assessment framework from the perspective of direct loss, which is legible and applicable to urban risk management fields. Secondly, this paper innovatively introduces the idea of VSL to monetize the loss of life from potential deaths caused by earthquakes by combining the assessment of economic loss and non-economic life loss, thereby enriching the VSL evaluation literature.

The following section reviews the fundamental concepts of risk assessment, disaster risk and disaster loss, as well as relevant methods and models used in research and practice. Section 3 introduces Xiamen, China as the site of our case study. Section 4 proposes the urban seismic risk assessment framework from the perspective of direct loss as well as a set of relevant evaluation methods and models, with reference to the improvement and integration of existing methods in risk assessment, economic appraisal, earthquake studies and the theory of life value. Empirical research of the proposed assessment framework and corresponding methods is then conducted to measure the urban seismic risk in Xiamen, China, and findings are presented in Section 5. The final section discusses the proposed urban seismic risk assessment framework critically and draws our main conclusions.

## 2. Literature Review

Although the concept of risk has different definitions in diverse fields such as insurance [16], economics [17], engineering [18] and disaster science [2,19], it is usually described from two aspects: consequences or losses and probability of occurrence. According to ISO 31000, risk refers to the effect of uncertainty on objectives and the level of risk. It can be assigned to a single risk or to a combination of risks and is estimated by considering and combining the consequences and likelihoods of an event [20]. According to this definition, risk can be represented by the expected value of consequences; that is, the product of the probability and consequence of a certain event. Similar to the definition of risk, natural disaster risk is described as the degree of expected loss due to certain natural phenomena that may occur within a given time period and certain area [20,21]. In several studies, disaster risks have also been estimated through the framework of hazards, exposure and vulnerability [22,23,24,25].

The idea of seismic hazard assessment in existing literature and practice can be classified into three categories. (1) Probabilistic analysis: Taking the basic assumption of random seismicity, probabilistic models for Poisson distribution are used to simulate and analyze the probability of earthquake occurrence with different intensities [26,27,28]. (2) Deterministic statistical analysis of historical data: Based on geological fault survey data and regional historical seismic data, regression models are used to analyze seismic risks [29,30,31]. (3) Identification of mainshock events: Due to the limited difference in the magnitudes of major earthquake events in each cycle incubation period in a particular seismic zone, the measurement of the hazards of each earthquake area can be determined according to the magnitude of its major earthquake [32]. The methods and conclusions in the Seismic Ground Motion Parameter Zonation Map of China (GB18306-2015) [33] show the three above-mentioned types of research ideas.

The research objects of seismic exposure and vulnerability analysis include infrastructure, population, properties and socioeconomic activities and their potential death rates, damage rates or fragility curves in diverse earthquake scenarios, and they are therefore also related to the potential range and degree of disaster loss. The expected loss or value of the consequences of a disaster consists of non-economic loss (loss of human lives, injuries and other sociocultural aspects) and economic loss, which is further divided into direct economic loss (mainly the value of property loss) and indirect economic loss (or long-term economic effects) [34]. Different types of losses can be estimated through diverse evaluation methods, such as property evaluation models, input–output models, computable general equilibrium models and some environment evaluation models. Literature on earthquake disaster losses originated from the basic research of the earthquake insurance industry in the 1930s, starting with Freeman’s studies [35] on the regional loss assessment of earthquake damage and insurance in the U.S. Some frameworks and models have been proposed to practically or theoretically assess earthquake economic loss, such as the European Risk-UE project, the Rapid Seismic Assessment Method proposed by JRA-3 (the Joint Research Activities-3), the GEM (Global Earthquake Model) and the Open-Quake-Engine application platform presented by OECD (the Organization for Economic Co-operation and Development), and the methodological framework of physical and socioeconomic vulnerability within the urban system proposed in the Seventh Framework of the European Union, etc.

Main approaches to estimate disaster loss include methods for assessing the status quo of vulnerability, statistical data modeling, remote sensing image recognition and onsite survey sampling etc. While vulnerability assessment and statistical data models are often used in ex-ante loss measurements and risk predictions, the latter two types of methods are more applicable in post-disaster evaluations of damages. In addition, based on historical disaster data and socioeconomic statistics, indirect or long-term economic effects can be estimated through modeling methods.

Earthquake vulnerability analysis of buildings and macro socioeconomic indicator-based loss analysis are two major approaches in the current ex-ante assessment of direct seismic economic loss. Yin [36] introduced a method to construct a seismic damage matrix and vulnerability curve for buildings according to their materials, structures, heights and design codes etc., as well as to predict the extent of damages and corresponding economic losses for different building types under different seismic intensities according to the ground-motion intensity distribution and existing building data. Developed by the U.S. government organizations FEMA (the Federal Emergency Management Agency) and NIBS (the National Institute of Building Sciences), the HAZUS97 software also estimates the potential economic losses from earthquakes based on building data, geological conditions, possible earthquake locations and other socioeconomic data [6,10,37]. Bi’s study [8] on the vulnerability of masonry structures used the peak ground acceleration as the input of earthquake ground motion and proposed a rapid assessment method for seismic loss as well as a determination of relevant parameters. More recently, methodologies for vulnerability (fragility) curves have also been applied to estimate seismic damage and the direct economic loss of buildings at community and municipal levels [38,39].

However, when carrying out earthquake economic loss prediction on a larger spatial scale, the above approach requires a detailed inventory database of the structures and facilities in the region, which is not always readily available. By collecting and analyzing the data records of global earthquake disaster losses, Chen et al. [40] studied the statistical correlation between the GDP loss rate and earthquake intensity and developed a model to predict earthquake disaster losses in each geographical unit cell through the empirical GDP loss rate, earthquake probability and localized socioeconomic data. This type of method has relatively clear data sources and simpler calculation processes, but the spatial accuracy of the prediction results is lower, making it a less effective guide for disaster prevention planning and facility preparation.

Loss of life is an unavoidable aspect of the consequences of a catastrophe. In terms of an earthquake, normally, the greater the seismic intensity, the more severe the damage to construction and the larger the number of deaths and injuries [41,42]. In addition to local population density and building structure types, other factors closely associated with earthquake casualties include ground motion intensity, local geological and geomorphic conditions, the distance to the seismic faulting location and the timing of earthquake occurrence, etc. [7,36,43,44,45,46,47,48]. The approaches to earthquake casualty estimation used in relevant studies can be divided into two major categories: one approach is to build statistical regression models based on the casualty data in plenty of historic earthquake records, with seismic intensity as a parameter, and then to figure out the formula and coefficient of the potential number or the probability of casualties [44,46,49,50,51,52]; the other method is based on the perspective of the seismic vulnerability of buildings, where casualty models are established according to population distribution, with the building destruction rate and number of collapses as the parameters [5,37,53,54,55].

However, major investigations into earthquake casualty estimation are limited to the modeling of the expected death rate or expected number of deaths due to earthquakes. There are always two dimensions in the definition and measurement of disaster loss, which are the economic loss and the number of casualties. In the current context of natural disasters, there are few studies which include the calculation and evaluation of human life losses and economic losses in the same dimension. On the one hand, in previous natural disaster risk analyses, the loss of life has been easy to ignore because it is difficult to monetize in the same way as property loss; on the other hand, due to technical or ethical reasons, there is still a gap in evaluating the loss of life value in disasters.

In daily life, the value of a single human life is not assigned a certain price; however, in scientific research, we should never deny or ignore the actual impact of disaster casualties on human society. In the theory of value of life, the value of a person’s life can be estimated through statistical methods, which is called the value of a statistical life (VSL) [13,14,56]. The evaluation methods for VSL, including human capital approaches [57] and willingness-to-pay approaches [14], which are often applied in policy evaluation practices in diverse regions [58,59,60], may bridge the gap between the study of life loss and disaster risk assessment. According to findings from existing estimates of VSL from human capital approaches and willingness-to-pay approaches, age has effects on the VSL [56], with age–VSL functions showing a significant inverted-U shape [61]. Compared with willingness-to-pay approaches, human capital approaches are easier to understand and collect data; however, the traditional human capital method only applies to people with income and does not apply to children, the elderly, self-sufficient farmers and self-employed groups. Yang improved the human capital approach by bringing factors of human capital input before death, expected lifetime income and spiritual loss caused by death into the estimation of VSL [62]. The monetization of the loss of life value, which is a vital part of non-economic loss, exemplifies innovative risk analysis with a scientifically rigorous and reasonable approach and helps us to understand both direct economic loss and life loss in a single context.

## 3. Site Identification and Data Source

**Figure 1:**
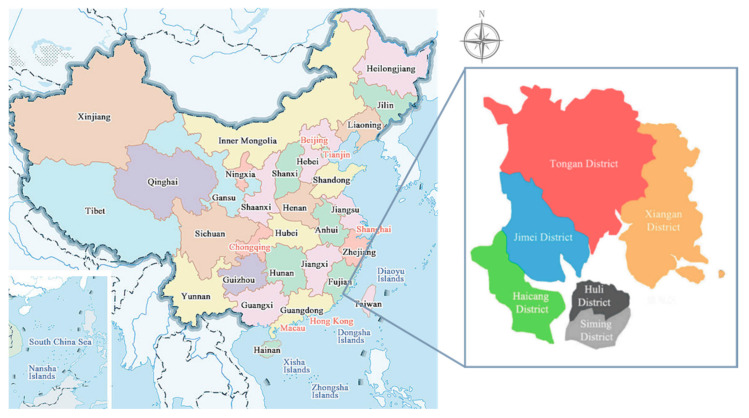
The location of Xiamen in China and the administrative divisions of the city.

Located on the southeast coast of Fujian Province in China (see Figure 1), Xiamen is one of the main central cities and harbors in China and is of political and economic importance. The scope of empirical research is the municipal administrative area of Xiamen, including the main island of Xiamen (Siming District and Huli District) and the other four coastal districts (Haicang District, Jimei District, Tong’an District and Xiang’an District). Xiamen has a resident population of more than 3.8 million. The city is situated in the middle of the Binhai Seismic Fault Zone (Changle-Zhao’an part) and 200~300 km west of the Western Taiwan Seismic Belt (Hsinchu-Kaohsiung part). As its geological structure determines its earthquake susceptibility, earthquakes of magnitude 6 or above have occurred nearby many times over the course of its history and caused varying degrees of damage to the area. Although no destructive earthquakes have been recorded in the Xiamen area, the fault zone still has strong activities, with obvious crustal movement. According to the Chinese code for the seismic design of buildings (GB50011-2010) [63] and the seismic ground motion parameter zonation map of China (GB18306-2015) [33], Xiamen has a seismic fortification intensity of VII degrees, and the peak horizontal ground acceleration is 0.15 g.

In line with the Masterplan of Xiamen (2017–2035) and the Urban Comprehensive Disaster Prevention Plan (2017–2035), this study adopts the “community unit” as its evaluation unit and takes this as the minimum data unit to collect and collate other socioeconomic data and building information for empirical analysis, which is a smaller scale than used in traditional seismic risk research at the urban level. Xiamen is divided into 93 community units, of which 17 are in Siming District, 11 are in Huli District, 14 are in Haicang District, 14 are in Jimei District, 15 are in Tong ’an District and 22 are in Xiang’an District. 

**Figure 2:**
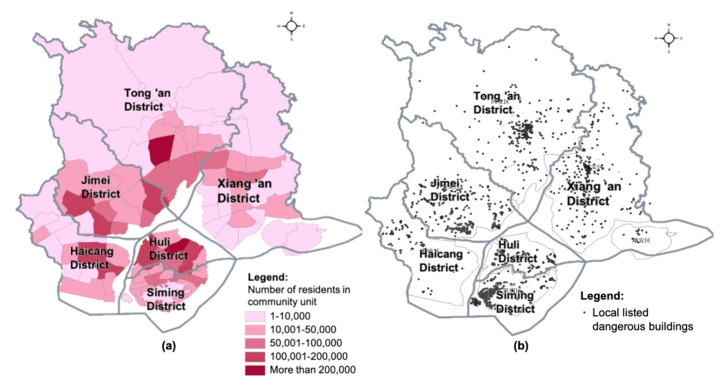
The distributions of population (**a**) and locally listed dangerous buildings (**b**) in community units in Xiamen.

Data acquisition and database establishment are the foundations of risk assessment. In this empirical study, we constructed a multi-source seismic risk database including traditional socioeconomic data as well as local building data (Table 1). Taking the community unit in Xiamen as the basic evaluation unit of this study, data registration and format conversion were carried out by relying on ArcGIS software (Geographic Information Systems, developed by Esri, USA). In terms of the population distribution in Xiamen, Huli District has the highest population density. There are also high numbers of residents in Siming District, coastal areas of Haicang District and Jimei District and central communities in Tong’an District and Xiang’an District, meaning that more people are exposed when an earthquake occurs (Figure 2a). In addition, according to local building survey data, there is a greater number of dangerous buildings in central communities in Tong’an District, Xiang’an District and communities especially in the east of Siming District, meaning that there is more vulnerable infrastructure facing an earthquake threat (Figure 2b).

## 4. Seismic Risk Assessment Methods from the Perspective of Direct Loss

### 4.1. Quantitative Evaluation Method of Disaster Risk

Risk can be presented as the product of the probability of an event and its corresponding quantified effects, namely the expected value of consequences. The concept of the expected value of loss represents how much is likely to be lost on average, and the expected value is calculated by the sum of the results of multiplying every possible outcome by its corresponding probability. Similarly, in this research, we aim to describe and measure the risk of one disaster through the framework of its expected value of impacts, which directly reflects both the likelihood of occurrences and the severity of corresponding negative consequences of a certain intensity of hazard.

More specifically, in combination with the definition of risk and the basic attributes of natural disasters, in this risk assessment framework, we consider one possible natural disaster faced by an area within a single-year period. The hazard risk of a certain intensity can thus be calculated as the product of its annual probability of occurrence and the direct loss, which is the annual expected value of direct loss in this disaster scenario. The overall risk of one disaster is the sum of the annual expected values of the impact of each disaster intensity scenario. Therefore, in seismic risk assessment practice, three steps in this framework should be followed: first, calculations of the annual probability of occurrence for each seismic intensity of the study site; then, estimations of the values of direct loss from each seismic intensity scenario; and finally, measurements of the annual expected value of direct loss in this disaster scenario and the overall seismic risk.

The probability of the occurrence of earthquakes of seismic intensity is calculated by analyzing the corresponding annual probability of exceedance. Disaster loss includes economic loss (direct and indirect) and non-economic loss. Due to the complex composition of indirect losses, without an authoritative and unified definition, and the fact that these are usually suffered over a longer period, this risk assessment framework focuses on the direct loss caused by one disaster. The concept of direct loss ($Loss\left(I\right)$) used in this research consists of the direct economic loss ($L_{DE}\left(I\right)$) and the loss of VSL ($L_{VSL}\left(I\right)$) (Equation (1)). Thus, the risk of a certain earthquake intensity ($Risk\left(I\right)$), namely the annual expected value of direct loss in this disaster scenario, can be calculated by the product of its probability and loss (Equation (2)).

(1) $$Risk\left(I\right)\;=\;Probability\left(I\right)\;\times \;Loss\left(I\right)$$

(2) $$Loss\left(I\right)=L_{DE}\left(I\right)+L_{VSL}\left(I\right)$$

Some conditions and limitations of this seismic assessment model are as follows: (1) it requires relatively complete building data and major socioeconomic data of a city; and (2) it takes into account only the direct loss (economic loss in building damage and loss of VSL) resulting from an earthquake and does not consider the value loss of indirect and long-term impacts of an earthquake in our measurement framework.

### 4.2. Calculation Methods of the Annual Probability of Earthquake Occurrence

The probability of exceedance refers to the chance of the occurrence of such hazards at a given earthquake ground motion or higher. The Poisson process for earthquake occurrence, which is a classic probabilistic seismic-hazard model [64], represents the homogeneous and spatially random characteristics of earthquake occurrence in a region. Based on Cornell’s Poisson Earthquake Occurrence Model, Gao derived the basic formula for calculating the probability of occurrence of earthquake intensity and simplified it [27,28].

In China, seismic fortification intensity refers to seismic intensity as the basis for a region’s seismic fortification according to the provisions of the state authority. The standard for the value of the seismic fortification intensity of a city is its basic ground motion intensity, which means that an area will exceed the earthquake intensity with a probability of 10% under general site conditions within the next 50 years. The specific value is based on the seismic fortification division in the seismic code (GB18306-2015) [33]; that is, in a 50-year period (which is the expected design life for a building), the earthquake intensity with a 10% probability of exceedance is defined as the seismic fortification intensity—i.e., $P_{50}\left(I,0\right)$ is equal to 10%. Thus, by checking the seismic fortification intensity of a city given by the seismic code [33], the probability of exceedance of this earthquake intensity in a one-year period can be calculated (Equation (3)).

(3) $$P_{1}\left(I,0\right)=1-{\left[1-P_{50}\left(I,0\right)\right]}^{\frac{1}{50}}$$

where $P_{1}\left(I,0\right)$ denotes the likelihood that an earthquake of fortification intensity occurs in one year and $P_{50}\left(I,0\right)$ denotes the probability of the designed basic ground motion and seismic fortification intensity (I) of a place, according to GB18306-2015 [33].

According to GB18306-2015 [33], since earthquakes with 63% and 2% probabilities of exceedance in a 50-year period are specified as frequent and rare, respectively [33], their possibility of occurrence in a one-year period can also be calculated as above (Table 2). In this empirical research in Xiamen—a city with a seismic fortification intensity of VII degrees—we will focus on risk analysis considering the disaster scenarios of three characteristic earthquakes, which are a frequent earthquake (a seismic intensity of VI degrees), an earthquake of fortification intensity (a seismic intensity of VII degrees) and a rare earthquake (a seismic intensity of VIII degrees).

### 4.3. Measurement Methods of Direct Economic Losses in Three Earthquake Scenarios

Historic earthquake records and damage analysis have shown that massive construction damages due to considerable ground motions are the major causes of economic losses and casualties. The direct economic losses in earthquake disasters mainly include two parts, which are structural losses (costs to repair or rebuild affected construction) and indoor property losses (the damage to the value of indoor fixed assets and equipment). Both the structure and indoor property losses are related to the extent of construction damage. Based on existing earthquake damage evaluation models [3,6,7,8,9,10,11,12] and in accordance with the Chinese code of classification of earthquake damage to buildings (GB/T24335-2009) [65] and GB18306-2015 [33]), in this research, the total direct economic losses for earthquakes of a certain intensity are measured with the following formulas (Equations (4)–(6)):

(4) $$L_{DE}{\left(I_{i}\right)}_{n}=L_{B}{\left(I_{i}\right)}_{n}+L_{P}{\left(I_{i}\right)}_{n}$$

(5) $$L_{B}{\left(I_{i}\right)}_{n}=\overset{K}{\underset{k=1}{\sum }}\overset{5}{\underset{j=1}{\sum }}P_{k}\left(D_{j}|I_{i}\right)\;\times \;l_{B}{\left(D_{j}\right)}_{k}\;\times \;{\left(S_{k}\right)}_{n}\;\times \;P_{k}$$

(6) $$L_{P}{\left(I_{i}\right)}_{n}=\overset{K}{\underset{k=1}{\sum }}\overset{5}{\underset{j=1}{\sum }}P_{k}\left(D_{j}|I_{i}\right)\;\times \;l_{p}{\left(D_{j}\right)}_{k}\;\times \;\beta {\left(S_{k}\right)}_{n}\;\times \;\mu P_{k}\;\times \;\gamma_{1}\;\times \;\gamma_{2}$$

where *n* denotes the nth evaluation unit, *n* ∈ N* (the total number of evaluation units); *j* denotes different levels of damage, *j* = 1, 2, 3, 4, 5; *k* denotes the *k*th category of a building structure, *n* ∈ K* (the total number of structure categories);$\;L_{DE}{\left(I_{i}\right)}_{n}$*,* $L_{B}{\left(I_{i}\right)}_{n}$*,* $L_{P}{\left(I_{i}\right)}_{n}$ denote the direct economic loss, structure loss and indoor property loss, respectively, in the *n*th evaluation unit due to an earthquake with an intensity of $I_{i}\;\mathrm{degrees}$; $\;P{\left(D_{j}|I_{i}\right)}_{k}$ denotes the damage ratio of the *k*th category of a building structure suffering *j* level of damage; $\;l_{B}{\left(D_{j}\right)}_{k}$ denotes the direct economic loss ratio of the *k*th category of a building structure with *j* level of damage; ${\left(S_{k}\right)}_{n}$ denotes the total construction surface area of all the *k*th category of building structures in the *n*th evaluation unit; $P_{k}$ denotes the replacement unit price of the main building structure; β denotes the ratio coefficient of the construction area of mid-to-high-end indoor decorations to the total construction surface area of the house; $l_{P}{\left(D_{j}\right)}_{k}$ denotes the direct economic loss ratio of indoor property of the *k*th category of building structures with *j* level of damage;  μ denotes the ratio coefficient between the unit price to replace the mid-to-high-end indoor decoration and the cost of the main structure, which represents the rate of cost losses of the indoor property caused by earthquake damage; and $\;\gamma_{1}\;$and $\gamma_{2}$ denote the correction coefficients for differences in economic development and different functions, respectively.

The five levels of damage—*j* = 1, 2, 3, 4, 5—refer to mainly intact, slightly damaged, moderately damaged, extensively damaged and completely damaged buildings, respectively. Building structures remain basically intact when earthquakes are less severe than their fortification. In accordance with GB50011–2010 [63], and with reference to the relevant literature from China’s Institute of Engineering Mechanics, National Seismological Administration [66,67,68], the following table presents the values of the damage ratio *(*$P{\left(D_{j}|I_{i}\right)}_{k}$ in this research (Table 3). More specifically, according to the second part of Table 3, for example, for building structures of a seismic fortification of intensity VII degrees facing an earthquake intensity of VI degrees, 85% of the buildings would be mainly intact and 15% slightly damaged; facing an earthquake intensity of VII degrees, 57% of the buildings would be mainly intact, 28% slightly damaged and 15% moderately damaged; facing an earthquake intensity of VIII degrees, 20% of the buildings would be mainly intact, 37% slightly damaged, 28% moderately damaged and 15% extensively damaged, and so on.

Different structure types have different aseismic capabilities. In the empirical study in Xiamen, the whole city has a seismic fortification intensity of VII degrees, meaning that the majority of urban buildings are designed and constructed according to the building codes, while there are still many existing private buildings distributed in informal urban villages. For structures built without following any specific seismic provisions, their aseismic capabilities are estimated to be no higher than a fortification intensity of VI degrees. Important public buildings, such as schools and hospitals, which are required to improve their degree of earthquake resistance, are estimated to be structures with a seismic fortification intensity of VIII degrees in Xiamen’s case. The values given in the rest of Table 3 thus provide the damage ratios for different structure categories with diverse aseismic capabilities facing different earthquake intensities.

Since this empirical research is conducted in a Chinese city, the values of other parameters in Equations (5) and (6), such as $l_{B}{\left(D_{j}\right)}_{k}$ and β, are estimated within the ranges given by the relevant Chinese Code (GB/T24335-2009) [65] and determined according to building types and local development status quo in Xiamen. The direct economic loss ratio $l_{B}{\left(D_{j}\right)}_{k}$ refers to the ratio of repair costs and replacement cost of the affected constructions, which is determined according to the structure type and local civil engineering situation and varies with the degrees of damage caused by an earthquake (Table 4). Table 5 illustrates the value ranges given in [65] for the ratio coefficient of a construction area of mid-to-high-end indoor decorations to the total construction surface area (β). With a resident population of more than 3.8 million, Xiamen is a metropolitan city, so the values of parameter β are determined and used in our calculation.

The size of decoration expenses is related to factors such as building functions, structures and economic development, etc., especially for those buildings with mid-to-high-end indoor decorations, where the value of property damage cannot be ignored. Relevant studies from the Institute of Engineer Mechanics, China Earthquake Administration [67,68,69,70] show that when the structural damage is slight, the loss ratio of indoor property is higher than that of the main structure. Furthermore, the difference between the indoor property loss ratio and the main structure loss ratio increases with the degree of damage to the main structure. Based on the conclusions of relevant literature [69,70,71], we organize the value ranges for the direct economic loss ratio of indoor property for different types of structures ($l_{P}{\left(D_{j}\right)}_{k}$) (Table 6). The ratio coefficient μ and correction coefficients $\gamma_{1}$ and $\gamma_{2}\;\;$help us to represent and adjust the differences in indoor property losses in diverse circumstances, such as different costs of interior decorations, different local economic development levels and different functions of buildings. Table 7 [72] shows the value ranges for the corresponding ratio coefficients between the unit price to replace the mid-to-high-end indoor decoration, the cost of main structure mid-to-high-end decoration and the unit price to replace the main structure (μ). In this research, the value ranges for the correction coefficients for differences in economic development ($\gamma_{1}$) and different functions ($\gamma_{2}$), respectively, are determined with reference to the post-earthquake field works listed in part 4 of the assessment of direct seismological loss (GB/T18208.4-2011) [72] (Table 8).

### 4.4. Measurement Methods of Loss of VSL in the Context of an Earthquake

The expected loss of VSL in an area relates to the expected number of deaths in earthquakes. Here, the research object of VSL is the direct and average impact of the death of local average people in all age groups. The total loss of VSL of all casualties in the context of a natural disaster can thus be calculated by multiplying the expected number of deaths in one earthquake and the average VSL of local residents.

(7) $$L_{VSL}{\left(I_{i}\right)}_{n}=VSL\times \mathrm{ND}{\left(I_{i}\right)}_{n}$$

where $L_{VSL}{\left(I_{i}\right)}_{n}$ and $\mathrm{ND}{\left(I_{i}\right)}_{n}\;$denote the loss of VSL and the number of deaths caused by earthquake intensity $I_{i}$ in the *n*th evaluation unit, respectively, and VSL denotes the average VSL of local residents in the context of the earthquake.

In evaluations using traditional approaches, individuals from different age groups have different VSLs, while they also show different vulnerabilities and probabilities to injury or loss of life in natural disaster scenarios. This study tries to improve the traditional human capital approach to cover the situation of life loss in all age groups when an entire local society faces a catastrophe. Without considering the distribution of mortality among ages in earthquakes of the same intensity, the loss of VSL caused by an “average local resident” who may die in an earthquake is monetized as the sum of total human capital inputs and total human capital income. The death age of this “average local resident” is the average age of the local population, whose life expectancy is the local average life expectancy. Thus, the loss of human capital includes the loss of the total human capital investment when there is no income, as well as the loss of future total income at the time of actual death. The following formulas (Equations (8)–(10)) explain the model used to evaluate the average VSL of local residents lost in an earthquake, which incorporates factors such as residents’ life expectancy, human capital investment and income, the number of years of wage income, years of education, the growth rate of human capital investment, the growth rate of human capital income and the discount rate, etc.

(8) $$VSL=VSL_{1}+VSL_{2}$$

(9) $$VSL_{1}=\;X+X\;\times \;\left(\frac{1+\alpha }{1+\beta }\right)+X\;\times \;{\left(\frac{1+\alpha }{1+\beta }\right)}^{2}+\cdots \cdots X\;\times \;{\left(\frac{1+\alpha }{1+\beta }\right)}^{i-1}$$

(10) $$VSL_{2}=Y+Y\;\times \;\left(\frac{1+\gamma }{1+a}\right)+Y\;\times \;{\left(\frac{1+\gamma }{1+a}\right)}^{2}+\cdots \cdots Y\;\times \;{\left(\frac{1+\gamma }{1+a}\right)}^{n-1}$$

where $VSL_{1}$ and $VSL_{2}$ denote total human capital investment and total human capital income respectively; X denotes human capital expenditure (including costs for education, medical care and life consumption); *Y* denotes human capital income (in traditional human capital models, one’s income represents one’s human capital); α, β and γ are, respectively, the discount rate, the growth rate of human expenditure in the past and the future growth rate of human capital, which are determined by using the average growth rates of expenditure and disposable income per capita in recent years; i denotes the number of years of human capital investment (generally, the number of average years of education or an average pre-working age with no earning capacity); and n denotes the number of years of lost income (calculated as the difference between average life expectancy and actual age of death).

Earthquake records and analysis show that the state of building damage is the most direct cause of casualties, making the number of deaths a function of the seismic vulnerability of building structures. With regard to the main factors in earthquake casualties, in this study, the model of potential population losses in an earthquake includes building damage states as the main parameters as well as population distribution, earthquake impact intensity, etc.

(11) $$\begin{array}{cc} \mathrm{ND}{\left(I_{i}\right)}_{n} & =\overset{4}{\underset{k=1}{\sum }}A_{3}{\left(D_{3}|I_{i}\right)}_{k,n}\;\times \;RD_{3}\;\times \;\rho_{n}\;\times \;{\left(f_{\rho }\right)}_{n}+\overset{4}{\underset{k=1}{\sum }}A_{4}{\left(D_{3}|I_{i}\right)}_{k,n}\;\times \;RD_{4}\;\times \;\rho_{n}\\ & +\overset{4}{\underset{k=1}{\sum }}A_{5}{\left(D_{5}|I_{i}\right)}_{k,n}\;\times \;RD_{5}\;\times \;\rho_{n} \end{array}$$

where $ND\left(I_{i}\right)$ denotes the expected number of deaths caused by an earthquake of intensity $I_{i}\;$in the *n*th evaluation unit; $A_{3}{\left(D_{3}|I_{i}\right)}_{k}$, $A_{4}{\left(D_{4}|I_{i}\right)}_{k}\;$and $A_{5}{\left(D_{5}|I_{i}\right)}_{k}$ denote the total areas of the *k*th category of building structures with moderate, extensive and complete damage states, respectively, caused by an earthquake of intensity $I_{i}\;$in the *n*th evaluation unit; $RD_{3}$, $RD_{4}$ and $RD_{5}$ denote the corresponding rates of death in building structures with moderate, extensive and complete damage states, respectively; and  ρ and$\;f_{\rho }$ denote the estimated indoor population density and correction factor.

With reference to similar research using building seismic vulnerability and earthquake intensity as main parameters [4,5], and based on the principles that “under the same earthquake intensity, the more serious the structural damage, the higher the death rate” and “with the same damage level, the greater the earthquake intensity, the higher the death rate”, the values of the rates of death in this research are shown in Table 9.

The indoor population density refers to the average number of people per unit area in buildings according to their use, time periods and regions, and it increases with higher local population density. In the empirical study in Xiamen, given a lack of data about the occupancy rates of each building during the day and at night, we use an average daytime building occupancy rate of 70% [5,73] to estimate the indoor population density. For evaluation units with a higher local population density, the indoor population density is higher in calculations. Thus, to estimate the indoor population density, this research uses the adjusted daytime indoor population density formula below (Equation (12)) with the density correction factor (usually a value of 0.8~1.2) shown in Table 10.

(12) $$\rho =\frac{7}{10}\times \frac{m_{n}}{A_{n}}\;\times \;w$$

where ρ denotes the indoor population density, $m_{n}\;\mathrm{and}\;A_{n}$ denote the total resident population and the total area of all types of buildings in the nth evaluation unit, and w denotes the correction factor of the estimated indoor population density.

## 5. Results

### 5.1. Direct Economic Loss Evaluation

**Figure 3:**
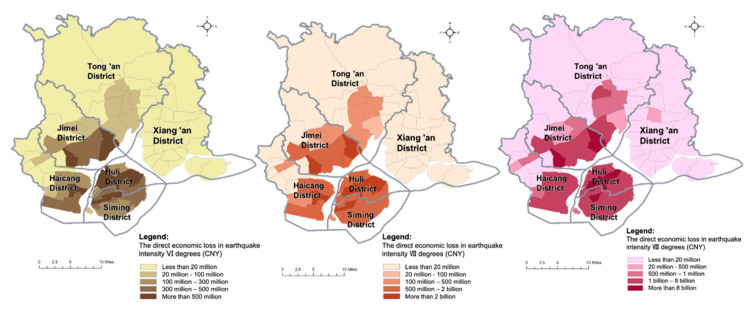
The distributions of direct economic losses in three earthquake scenarios.

Through the calculation formulas and parameter values provided in Section 4.2, the direct economic loss in each evaluation unit is measured, respectively, assuming earthquakes with intensities of VI degrees, VII degrees and VIII degrees in Xiamen. The distributions of the direct economic loss in three earthquake scenarios are visualized by ArcGIS software (Figure 3). The direct economic loss in each evaluation unit increases with the rise in earthquake intensity. According to the calculation results, the expected total direct economic losses amount to about 17.5 billion (CNY) (2.5% of the total value) when the earthquake intensity is VI degrees (a frequent earthquake), 64.9 billion (CNY) (9.4% of the total value) when the earthquake intensity is VII degrees (an earthquake of fortification intensity), and 223.6 billion (CNY) (32.4% of the total value) when the earthquake intensity is VIII degrees (a rare earthquake) in Xiamen. Major economic losses are expected to be concentrated in Siming District, Huli District and coastal areas of Haicang District and Jimei District due to dense urban development and economic activities. Several evaluation units with the highest direct economic loss in earthquakes are found to have a number of urban villages with lower construction standards, making them difficult to protect in severe disasters and leading to a great number of casualties and large economic losses, even in an earthquake of fortification intensity.

### 5.2. Loss of VSL in an Earthquake

Relevant socioeconomic data were collected from government open-source statistics including the Special Economic Zone Yearbooks of Xiamen (2015–2018), Xiamen’s 13th Five-Year Education Development Plan (2017) and Xiamen’s 2018 National Economic and Social Development Statistical Bulletin (2018). The city’s average life expectancy is 80.75 years and the average age of the population is about 33. Using Equations (8)–(10) in Section 4.4, the parameters are measured one by one. The value of human capital expenditure (X) adopts a per capita expenditure of 33,192 CNY in Xiamen in 2018; the value of the growth rate of human expenditure in the past (β) takes the average annual growth rate of human expenditure in the past four years as a relatively fair value for this, which is 6.7%; the value of human capital income (*Y*) adopts the per capita disposable income of 50,948 CNY in Xiamen in 2018; the value of the future growth rate of human capital (γ) takes the average annual growth rate of the per capita disposable income in the past four years as a relatively fair value for this, which is 8.4%; the discount rate (α) is valued at 6% in this case as it is often valued around 5%–7% in the human capital literature; and the number of years of human capital investment (i) is 19 and the number of years of lost income (n) is 48 in this case study. Thus, the average loss of VSL caused by the death of residents in an earthquake (or other disasters) is 4,935,500 CNY per capita in Xiamen.

**Figure 4:**
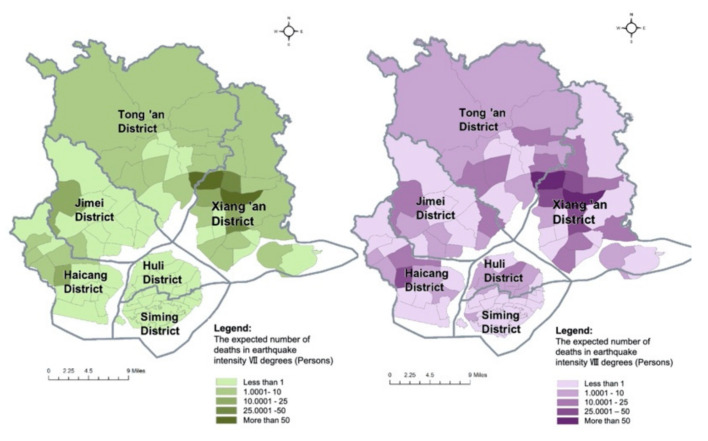
The distributions of the expected number of deaths in earthquake scenarios with intensities of VII degrees and VIII degrees.

Using Equation (11) and the values of parameters provided in Section 4.4, the number of deaths in each evaluation unit is measured, respectively, in situations of earthquake intensities of VI degrees, VII degrees and VIII degrees in Xiamen. The distributions of the number of deaths in three earthquake scenarios are visualized by ArcGIS software. The expected number of deaths in each evaluation unit increases with the rise in earthquake intensity. According to the calculation results of the empirical study in Xiamen, three people on average may die in an earthquake with an intensity of VI degrees (frequent earthquake), 367 people on average may die in an earthquake with an intensity of VIII degrees (rare earthquake). As for the estimated fatalities in a frequent earthquake in Xiamen, the values are below 1 in all the evaluation units, indicating that the life safety issue can be basically guaranteed in this scenario. In some areas such as central Xiang’an District, poor housing quality might lead to higher casualties, even in a frequent earthquake. Using Equation (7), the loss of VSL in each evaluation unit is measured, respectively, in situations of earthquakes with intensities of VII degrees and VIII degrees in Xiamen. Their distribution patterns are similar to the distributions of the number of deaths in the three earthquake scenarios shown in Figure 4.

### 5.3. Seismic Risk Assessment

**Figure 5:**
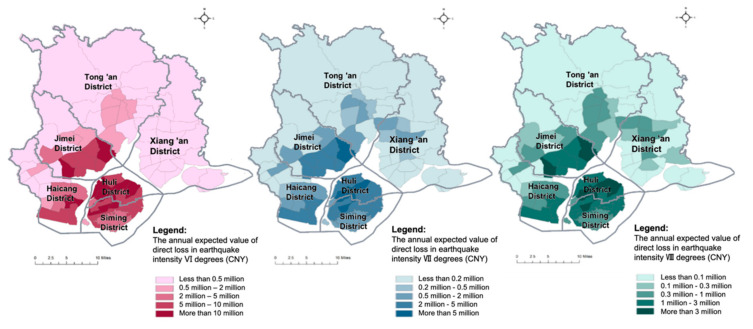
The seismic risk distribution (the distributions of the annual expected value of direct loss) in three earthquake scenarios.

Using Equations (1) and (2) provided in Section 4.2 and with the calculation results given in Section 5.1 and Section 5.2, the seismic risks, the annual expected values of direct losses in the three earthquake scenarios with intensities of VI degrees, VII degrees and VIII degrees in Xiamen are measured, respectively. Considering the different probabilities of a frequent earthquake, an earthquake of fortification intensity and an earthquake of rare intensity, their risk values are different. Figure 5 shows their spatial risk distributions, as visualized by ArcGIS software. Xiamen faces a higher seismic risk from an earthquake intensity of VI degrees than that of higher intensities. Coastal community units in Jimei Distrit, Haicang District and Huli District experience a relatively high risk in earthquake scenarios.

Since the damage from lighter earthquakes is negligible and the probability of occurrence of greatest severity is too small to consider, in order to present the overall seismic risk in Xiamen, we can further sum up the results of annual expected values of direct loss of the above three earthquake scenarios in each evaluation unit through the following formula.

(13) $${\left(R_{Seismic}\right)}_{n}=\overset{8}{\underset{I_{i}=6}{\sum }}\left(L_{DE}{\left(I_{i}\right)}_{n}+L_{VSL}{\left(I_{i}\right)}_{n}\right)\;\times \;P\left(I_{i}\right)$$

where ${\left(R_{Seismic}\right)}_{n}$ denotes the quantitative seismic risk in the *n*th evaluation unit; $L_{DE}{\left(I_{i}\right)}_{n}$ and $L_{VSL}{\left(I_{i}\right)}_{n}$ denote the direct economic loss and the loss of VSL, respectively, in the *n*th evaluation unit due to an earthquake with an intensity of $I_{i}\;\mathrm{degrees}$; and $P\left(I_{i}\right)$ denotes the probability of occurrence of an earthquake with an intensity of $I_{i}\;$degrees.

**Figure 6:**
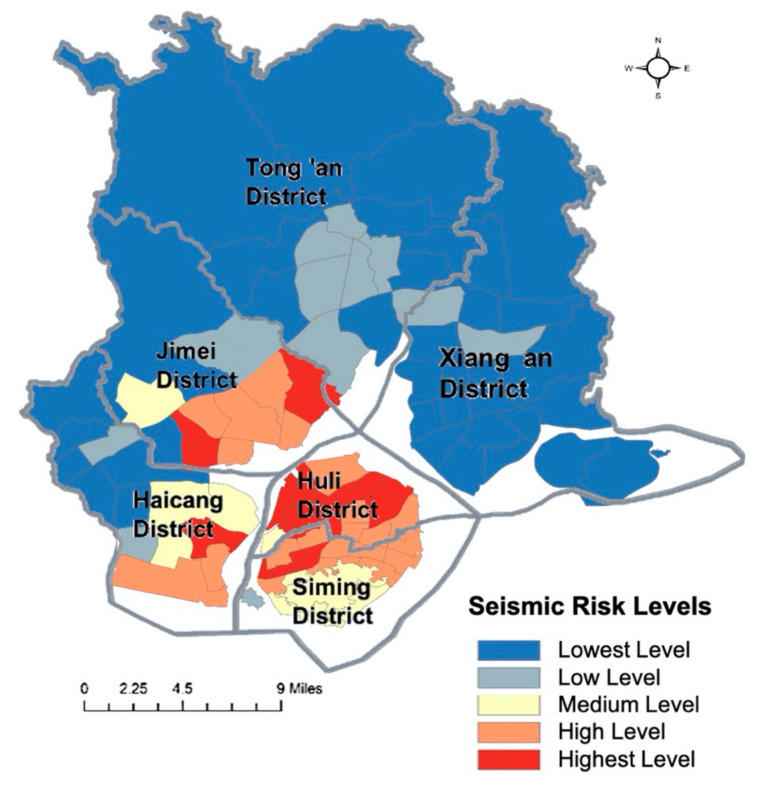
The overall seismic risk distribution of Xiamen.

Then, we use the natural fracture (Jenks) method to reclassify the evaluation results of seismic risk in Xiamen and visualize these with ArcGIS software (Figure 6). Taking the probabilities of a frequent earthquake, an earthquake of fortification intensity and a rare earthquake (earthquake intensities of VI degrees, VII degrees and VIII degrees, respectively) into consideration in seismic risk evaluation, 10 community units face the highest level of seismic risk, of which five are in Huli District, two are in Siming District, two are in Jimei District and one is in Haicang District, while all the community units in Tong’an District and Xiang’an District have the lowest or low levels of seismic risk and are thus relatively safe in earthquake scenarios. 

The overall seismic risk distribution of Xiamen shows a similar pattern to the urban development intensity. In the northern part of Xiamen, where there are mountains with limited urban development, the annual expected value of direct loss from earthquakes may reasonably be expected to be the lowest. The seismic risk is higher for communities with more densely developed urban infrastructure in the traditional central city areas (Huli District, Siming District and coastal areas of Haicang District and Jimei District) and with listed dangerous buildings concentrated in informal urban villages. In order to reduce the influence of earthquake occurrence on society, the following actions should be considered, especially for those community units designated with the higher and highest levels of seismic risk: (1) plan and prepare enough earthquake shelters for residents; (2) renovate and reinforce dilapidated buildings; and (3) improve disaster prevention education and evacuation drills. 

## 6. Discussion

This research was undertaken to design a new urban seismic assessment framework and practice in our case study. In combination with the definition of risk and the basic attributes of natural disasters, we aimed to quantitatively describe disaster risk through the measurement of the expected value of impacts, which directly reflects both the likelihood of occurrence and the severity of corresponding negative consequences. The proposed urban seismic risk assessment framework consists of three main parts: (1) calculating the annual probability of occurrence of different earthquakes is the starting point—calculations of recurrence rates in a one-year period for the three characteristic earthquakes in Xiamen are a backward extrapolation process based on the known conditions provided in relevant design codes [33,63,65,72]; (2) estimating the direct loss of each seismic intensity scenario is the major contribution of this risk analysis framework, including the evaluations of potential direct economic losses caused by building damage in an earthquake, the loss of the value of a statistical life (VSL), as measured by one average local resident death in a disaster, and predictions of potential earthquake casualties. In particular, the expected number of deaths in earthquakes that are calculated in this framework specifically represents the certain probability of death for residents, or the potential threat to lives caused by the earthquake. For example, if it is expected that 20 people might die in one evaluation unit in an earthquake according to the projection model, this number of 20 does not refer to a specific group of residents, and it is not known which 20 residents would die. Furthermore, this framework has improved the traditional human capital approach to evaluate the loss of VSL in disasters by expanding the concept of human capital income and defining the number of years of loss of human capital income as the difference between life expectancy and the actual age of death in order to cover all groups in society; (3) a seismic risk of a certain intensity can thus be calculated as the product of its annual probability of occurrence and the direct loss (the sum of the direct economic loss and the loss of VSL), which is the annual expected value of direct loss in this disaster scenario. The urban seismic risk assessment result is therefore the measurement of the annual expected direct loss in each of the three characteristic earthquake scenarios in Xiamen and then the summation of the seismic risks in each evaluation unit to further calculate the overall seismic risk distribution in Xiamen.

The experimental results provide substantial evidence for our original hypothesis, posed at the beginning of this study, that a seismic risk of a certain degree of intensity can be calculated through the measurement of the annual expected value of direct loss within our proposed assessment framework. By applying the proposed evaluation methods in all of the community units in Xiamen, the possible scope and extent of earthquake risks are determined and quantified. The results are also partially consistent with our second hypothesis that the overall seismic risk distribution pattern is strongly correlated with local building fragility in an earthquake as well as the distribution of construction. 

Unlike the approach used by the HAZUS system, which also reflects the perspective of disaster loss, our study estimates urban seismic risk by monetizing and combining the loss from building damage with the loss of life value resulting from earthquake casualties. The advantage of this urban seismic risk assessment framework lies in the fact that, through the calculation of the “annual expected direct loss”, it offers an effective quantitative measure of disaster risk by considering both the consequences and the probability of occurrence of disasters. Specifically, it includes the three following basic features:

1. A framework based on the basic concept of risk, with a legible structure to conceptualize the risk of disaster. With relevant data, this framework is thus easily applicable in all kinds of evaluation units, ranging from the regional and national scale to the municipal, street and even community scale, as well as urban risk assessment for other disaster scenarios. In line with relevant building codes and the parameter range given in this paper, the urban seismic risk assessment can be practiced in the case of relatively complete building data and major socioeconomic data, which are often open-source and can be obtained on the Internet or from public administration (see Table 1).
2. Through the measurement of the annual expected direct loss, disaster risks can be compared in different scenarios, locations and time periods. This is also applicable to the calculation the comprehensive risk of multi-hazard disasters by accumulating the results of the annual expected direct loss of single disasters for each evaluation unit and further analyzing the comprehensive risk distribution. It should be noted that, in our earthquake risk assessment models and empirical analysis, only the most important building and indoor property losses representing economic losses have been considered at the current stage. In the face of earthquakes with high intensities, the economic impacts resulting from earthquake secondary disasters and the damage of the urban lifeline infrastructure cannot be ignored. Meanwhile, for other urban disasters such as floods and fires, the forms and extents of economic losses need to be discussed separately.
3. This disaster risk assessment framework considers loss of life to be as important as economic impacts caused by disasters. The idea of VSL is innovatively introduced to monetize the life loss of potential deaths caused by earthquakes so that the direct consequences of earthquakes (both economic loss and non-economic life loss) can be compared and calculated in a single measurement. The improvement in approaches to estimating the loss of VSL in disasters also enriches the VSL evaluation literature and is conducive to the formulation of well-targeted disaster risk management, disaster relief actions and insurance policies and the evaluation of investment in disaster prevention and mitigation projects. However, according to our empirical study, for most urban earthquake scenarios (frequent earthquake, basic ground motion intensity earthquake and rare earthquake), the total loss in VSL is often smaller than the direct economic loss resulting from building damage, meaning that the effects of the distribution pattern of resident population on the overall seismic risk distribution is less than the building distribution patterns. This is partially because our estimate of earthquake casualties is delivered based on building destruction. Furthermore, among the VSL evaluation literature, the estimated value of life measured by the willingness-to-pay method is much higher than the value measured by the human capital method [14], which helps to explain the relatively small share of monetized loss of life in the total direct loss of a disaster.

## 7. Conclusions

This paper has reviewed the fundamental concepts of risk assessment, disaster risk and disaster loss and the relevant methods and models used in research and practice. Aiming to estimate, quantify and visualize the earthquake risk in each unit of the urban space, we have proposed an urban seismic risk assessment framework from the perspective of direct loss with a set of evaluation models and carried out an empirical study in Xiamen, China. Key findings of the case study include the expected direct economic losses and the expected number of deaths in three characteristic earthquakes in Xiamen, their estimated spatial distributions, the average VSL of local residents (in the disaster context), the overall seismic risk level of each urban community unit and spatial risk distributions. Admittedly, the proposed seismic risk framework only considers the direct consequences of earthquakes instead of the long-term indirect impacts, which include affected economic activities, sociocultural loss, costs of disaster relief and injuries etc. This study has provided a new perspective to understand and measure urban disaster risk while considering both occurrence frequencies and consequences. Future studies in this area will try to incorporate and quantify more types of disaster consequence to make the risk evaluation more precise and closer to reality. On the other hand, further empirical research will be able to practice risk evaluation of other disasters as well as the comprehensive risk assessment of multi-hazard disasters within the proposed disaster risk assessment framework for measuring annual expected direct losses.

## Figures and Tables

**Table 1:** Type of data and Sources.

Type of Data	Data Source
Population data	Statistical Yearbook and Economic Census Data of Xiamen
Socioeconomic data	
Population of each community unit Survey data of local listed dangerous buildings	Xiamen Urban Design and Planning Institute
Building structure types	
Land use data	Xiamen Municipal Bureau of Land Resources and Urban Planning
Administrative division boundary data	
Building location information Number of building floors	Open data from Baidu map API (Application Programming Interface) (http://lbsyun.baidu.com )
Construction area	
Building age information	National meteorological science data sharing service platform (http://data.cma.cn/)

**Table 2:** Annual probabilities of three characteristic earthquakes occurring in a one-year period in Xiamen.

Characteristic Earthquakes (Intensity Degrees)	Definition in GB18306–2015	Probability of Exceedance in One Year
Frequent earthquake (seismic intensity of VI degrees)	63% probability of exceedance in a 50-year period	$P\left(I_{6}\right)=$0.019688642
Earthquake of fortification intensity (seismic intensity of VII degrees)	10% probability of exceedance in a 50-year period	$P\left(I_{7}\right)=$0.002104992
Rare earthquake (seismic intensity of VIII degrees)	2% probability of exceedance in a 50-year period	$P\left(I_{8}\right)=$0.000403973

**Table 3:** The values of damage ratios for different structure fortifications with diverse seismic fortification intensities facing different earthquake intensities (unit: %).

Seismic Fortification Intensity	Damage Ratio	Earthquake Intensity				
		VI	VII	VIII	IX	X
**Structures with a seismic fortification intensity of VI degrees**	$D_{1}$—Mainly intact	57	20	5	0	0
	$D_{2}$—Slight damage	28	37	15	5	0
	$D_{3}$—Moderate damage	15	28	27	30	33
	$D_{4}$—Extensive damage	0	15	28	37	30
	$D_{5}$—Complete damage	0	0	15	28	37
	**Damage Ratio**	**Earthquake Intensity**				
**Structures with a seismic fortification intensity of VII degrees**		**VI**	**VII**	**VIII**	**IX**	**X**
	$D_{1}$—Mainly intact	85	57	20	5	0
	$D_{2}$—Slight damage	15	28	37	15	5
	$D_{3}$—Moderate damage	0	15	28	27	30
	$D_{4}$—Extensive damage	0	0	15	28	37
	$D_{5}$—Complete damage	0	0	0	15	28
	**Damage Ratio**	**Earthquake Intensity**				
**Structures with a seismic fortification intensity of VIII degrees**		**VI**	**VII**	**VIII**	**IX**	**X**
	$D_{1}$—Mainly intact	100	85	57	20	5
	$D_{2}$—Slight damage	0	15	28	37	15
	$D_{3}$—Moderate damage	0	0	15	28	27
	$D_{4}$—Extensive damage	0	0	0	15	28
	$D_{5}$—Complete damage	0	0	0	0	15

**Table 4:** The value ranges for direct economic loss ratios of different types (unit: %) [65].

	Direct Economic Loss Ratio	Level of Damage				
Building Types		$D_{1}\mathrm{Mainly}\;\mathrm{Intact}$	$D_{2}\mathrm{Slight}\;\mathrm{Damage}$	$D_{3}\mathrm{Moderate}\;\mathrm{Damage}$	$D_{4}\mathrm{Extensive}\;\mathrm{Damage}$	$D_{5}\mathrm{Complete}\;\mathrm{Damage}$
**Reinforced concrete and masonry buildings**	Value range	0–5	6–15	16–45	46–100	81–100
	Intermediate value	3	11	31	73	91
**Industrial plants**	Value range	0–4	5–16	17–45	46–100	81–100
	Intermediate value	2	11	31	73	91
**Urban bungalows and rural houses**	Value range	0–5	6–15	16–40	41–100	71–100
	Intermediate value	3	11	28	71	86

**Table 5:** The value ranges for the ratio coefficient of a construction area of mid-to-high-end indoor decorations to the total construction surface area of the building (unit: %) [65].

Urban Size		Building Types	
		Reinforced Concrete Structure	Masonry Buildings
Metropolitan cities Population ≥ 1 million	Value range	31–55	12–35
	Intermediate value	43	19
Medium-sized cities Population 200,000–1 million	Value range	17–35	5–11
	Intermediate value	26	8
Small cities Population ≦ 200,000	Value range	8–15	2–5
	Intermediate value	12	4

**Table 6:** Value ranges for the direct economic loss ratios of indoor property in different structure types (unit: %).

Building Types	Level of Damage				
	*D*_1_ Mainly Intact	*D*_2_ Slight Damage	*D*_3_ Moderate Damage	*D*_4_ Extensive Damage	*D*_5_ Complete Damage
Reinforced concrete buildings	2–10	11–25	26–60	61–90	91–100
Masonry buildings	0–5	6–19	20–47	48–85	86–100

**Table 7:** Value ranges for the ratio coefficients between the unit price to replace mid-to-high-end indoor decoration and the cost of the main structure (unit: %).

Urban Size		Building Types	
		Reinforced Concrete Structure	Masonry Buildings
Metropolitan Population ≥ 1 million	Value range	26–48	20–34
	Intermediate value	37	27
Medium-sized cities (Population 200,000–1 million)	Value range	19–38	16–25
	Intermediate value	29	21
Small cities (Population ≦ 200,000	Value range	15–30	10–20
	Intermediate value	23	15

**Table 8:** Value ranges for correction coefficients for differences in economic development levels ($\gamma_{1}$) and building functions (unit: %).

**Economic Development**	**Developed**	**Relatively Developed**	**Ordinary**
$\gamma_{1}\;$ ^1^	1.3	1.15	1.0
**Building Function**	**Residential**	**Educational and Health**	**Public**
$\gamma_{2}\;$ ^2^	1.0–1.1	0.8–1.0	1.1–1.2

In the empirical study in Xiamen, based on the city’s per capita GDP of 115,359 CNY in 2018, ranking among the top 20 Chinese cities (and highest in Fujian Province), ^1^ Xiamen can be considered an economically “developed” region, and thus $\gamma_{1}$ is valued at 1.3 in this case. ^2^ In the empirical study in Xiamen, the value of$\;\gamma_{2}$ is applied according to the land use of each evaluation unit.

**Table 9:** The predicted rates of death in buildings with different levels of damage under three earthquake scenarios.

Level of Damage		*D*_3_ Moderate Damage	*D*_4_ Extensive Damage	*D*_5_ Complete Damage
**Rate of death**	Frequent earthquake	1 × 10^−6^	8 × 10^−5^	2 × 10^−3^
	Earthquake of fortification Intensity	1 × 10^−5^	5 × 10^−4^	8 × 10^−3^
	Rare earthquake	3 × 10^−5^	8 × 10^−4^	2 × 10^−2^

**Table 10:** Value of the density correction factor of the estimated indoor population density [73].

Local Population Density (Unit: Persons/km^2^)	<50	50–200	200–500	>500
Value of correction factor	0.8	1.0	1.1	1.2

## References

[1] Pavlovic N. Seismic hazard and risk analysis. Proceedings of the Asia 2004 Conference on Earthquake Engineering.

[2] Varnes D.J. (1984). Landslide Hazard Zonation: A Review of Principles and Practice.

[3] Whitman R.V. (1973). Damage probability matrices for prototype buildings. Massachusetts.

[4] Anagnostopoulos S.A., Whitman R.V. On human loss prediction in building during earthquakes. Proceedings of the Sixth World Conference on Earthquake Engineering.

[5] Yin Z. (1992). Prediction of Earthquake Damage.

[6] Schneider P.J., Schauer B.A. (2007). HAZUS-its development and its future. Nat. Hazards Rev..

[7] Guo A., Hou S., Li H., Ou J. (2007). Earthquake loss prediction method for typical urban buildings II: Earthquake loss estimation. Earthq. Eng. Eng. Vib..

[8] Bi K. (2009). Vulnerability Analysis of Group Buildings and Rapid Assessment of Earthquake Losses. Ph.D. Thesis.

[9] Sun L. (2016). Urban Earthquake Damage Assessment Methods and System Development Research. Ph.D. Thesis.

[10] FEMA, NIBS (1997). Earthquake Loss Estimation Methodology-HAZUS97: Technical Manual.

[11] Cagnan Z., Sesetyan K., Zulfikar C., Demircioglu M.B., Kariptas C., Durukal E., Erdik M. (2008). Development of earthquake loss map for Europe. J. Earthq. Eng..

[12] Erdik M., Sesetyan K., Demircioglu M. (2010). Rapid earthquake hazard and loss assessment for Euro-Mediterranean region. Acta Geophys..

[13] Charles F. (1969). The Value of life. Harv. Law Rev..

[14] Schelling T.C., Chase S.B. (1968). The life you save may be your own. Problems in Public Expenditure and Analysis.

[15] Yu X., Tang Y., Liu C. (2009). Theoretical research on disaster life Value assessment. China Saf. Sci. J..

[16] Helm P. (1996). Integrated risk management for natural and technological disasters. Tephra.

[17] Knight F.H. (1921). Risk, uncertainty, and profit. Soc. Sci. Electron. Publ..

[18] UNEP (2002). Global Outlook 3: Past, Present and Future Perspectives.

[19] UNISDR (2009). UNISDR Terminology on Disaster Risk Reduction.

[20] ISO31000: 2018 (2018). Risk Management—Principles and Guidelines.

[21] O’Riordan T., Kates R.W., Burton I. (1986). Coping with environmental hazards. Geography, Resources, and Environment.

[22] IPCC (2014). Climate Change: Impacts, Adaptation, and Vulnerability. Part A: Global and Sectoral Aspects.

[23] Merz B., Kreibich H., Thieken A., Schmidtke R. (2004). Estimation uncertainty of direct monetary flood damage to buildings. Nat. Hazards Earth Syst. Sci..

[24] Mileti D.S. (1999). Disaster by Design: A Reassessment of Natural Hazards in the United States.

[25] Okada N., Tatano H., Hagihara Y. (2003). Integrated research on methodological development of urban diagnosis for risk and its applications. Disaster Prev. Res. Inst. Annu..

[26] Johnston A.C., Nava S.J. (1985). Recurrence rates and probability estimates for the New Madrid Seismic Zone. J. Geophys. Res. Solid Earth.

[27] Gao M. (1988). Discussion on the annual average incidence of earthquakes. Int. J. Seism. Dyn..

[28] Gao M. (1996). Probability model of earthquake intensity based on Poisson distribution. China Earthq..

[29] Junji K., Kenzo T., Tadanobu S., Kazuya F. (1991). Seismic risk analysis for the Osaka bay area on active fault data and historical earthquake data. Vol. Annu. Symp. Soc. Civ. Eng..

[30] Grapes R., Downes G. (1997). The 1855 Wairarapa, New Zealand, earthquake: Analysis of historical data. Bull. N. Z. Natl. Soc. Earthq. Eng..

[31] Qi Y., Jin X. (2009). Seismic risk analysis method based on historical records of earthquakes. Earthq. Disaster Prev. Technol..

[32] Qin S., Yang B., Wu X. (2015). Main events in the earthquake zone of mainland China knowledge I. Prog. Geophys..

[33] GB18306-2015 (2015). Seismic Ground Motion Parameter Zonation Map of China.

[34] Ma Z. (1990). Natural Disasters and Disaster Mitigation: 600 Queries and Answers.

[35] Freeman J.R. (1932). Earthquake Damage and Earthquake Insurance.

[36] Yin Z. (1996). Methods of Earthquake Disaster and Loss Prediction.

[37] Kircher C.A., Nassar A.A., Kustu O., Holmes W.T. (1997). Development of building damage functions for earthquake loss estimation. Earthq. Spectra.

[38] Kappos A. (2013). Seismic vulnerability and loss assessment for buildings in Greece. Seismic Vulnerability of Structures.

[39] Wiebe D.M., Cox D.T. (2014). Application of fragility curves to estimate building damage and economic loss at a community scale: A case study of Seaside, Oregon. Nat. Hazards.

[40] Chen Q., Chen Y., Chen L., Liu J., Li M. (1999). Estimation of earthquake losses by using macroeconomic approach. Chin. Sci. Bull..

[41] Nichols J.M., Beavers J.E. (2003). Development and Calibration of an earthquake fatality function. Earthq. Spectra.

[42] Li Y. (2009). Study on Earthquake Death in Yunnan. Ph.D. Thesis.

[43] Fu Z., Jiang L., Li G. (1994). Analysis of spatiotemporal strong distribution characteristics of life loss in earthquake disasters. Earthquake.

[44] Badal J. (2005). Vazquez-Prada, M.; Gonzalez, A. Backward quantitative assessment of earthquake and damages. Nat. Hazards.

[45] Hu Y. (2006). Earthquake Engineering.

[46] Pai C., Tien Y. (2007). A study of the human-fatality rate in near-fault regions using the Victim Attribute Database. Nat. Hazards.

[47] Zou K., Liu K., Li Y., Du L., He J. (2008). Systematic evaluation of risk factors for earthquake casualties. Chin. J. Evid.-Based Med..

[48] Liu G., Li G., Zhang X., Wie Z. (2015). Study on regional characteristics of life loss in Earthquakes in Yunnan Province 1. Earthq. Disaster Prev. Technol..

[49] Chen Q., Chen L. (1997). Prediction and assessment of earthquake disaster loss using GDP and population data. Acta Seismol. Sin..

[50] Wang X., Ding X., Wang L. (2009). A Study on the rapid assessment of disaster losses in Wenchuan Earthquake with magnitude 8 degrees. J. Seismol..

[51] Li J., Su J., Li X. (2011). Study on casualty assessment model of regional earthquake disasters. Henan Sci..

[52] Jaiswal K.S., Wald D.J., Earle P.S., Porter K.A., Hearne M. (2011). Earthquake casualty models within the USGS Prompt Assessment of Global Earthquakes for Response (PAGER) System. Human Casualties in Earthquakes.

[53] Liu X., Li H., He J. (1985). Earthquake damage estimation and economic decision-making model. Earthq. Eng. Eng. Vib..

[54] Ma Y., Xie L. (2000). Methods for estimating casualties in earthquakes. Earthq. Eng. Eng. Vib..

[55] Gao H., Li Q. (2010). Rapid assessment model of casualties in earthquake. J. Catastrophol..

[56] Viscusi W.K., Aldy J.E. (2003). The Value of a statistical life: A critical review of market estimates throughout the world. J. Risk Uncertain..

[57] Grossman M. (1972). On the concept of health capital and the demand for health. J. Political Econ..

[58] de Blaeij A., Florax R.J., Rietveld P., Verhoef E. (2003). The value of statistical life in road safety: A meta-analysis. Accid. Anal. Prev..

[59] Madheswaran S. (2007). Measuring the value of statistical life: Estimating compensating wage differentials among workers in India. Soc. Indic. Res..

[60] Viscusi W. (2012). What’s to know? Puzzles in the literature on the value of statistical life. J. Econ. Surv..

[61] O’Brien J. (2018). Age, autos, and the value of a statistical life. J. Risk Uncertain..

[62] Yang Z. (2010). Theoretical Method and Empirical Research on Life Value Assessment. Ph.D. Thesis.

[63] GB50011-2010 (2010). Chinese Code for Seismic Design of Buildings.

[64] Cornell C.A. (1968). Engineering Seismic Risk Analysis. Bull. Seismol. Soc. Am..

[65] GB/T24335-2009 (2009). Chinese Code of Classification of Earthquake Damage to Buildings.

[66] Xie L., Zhang X., Zhou Y. (1996). On the seismic fortification standard of engineering. Earthq. Eng. Eng. Vib..

[67] Zhang F., Xie L., Fan L. (2004). Prediction of earthquake losses of urban Structures. Earthq. Eng. Eng. Vib..

[68] Zhang J., Pan W., Song Z., Lin H., Wu X. (2017). Seismic vulnerability assessment of urban buildings based on intensity difference. Earthq. Eng. Eng. Vib..

[69] Chen H., Sun B., Sun D. (2010). A study on the ratio of renovation damage to economic damage assessment in earthquake. Earthq. Disaster Prev. Technol..

[70] Chen H. (2008). Study on Assessment Methods of Earthquake Damage in Urban Housing Construction and Decoration. Ph.D. Thesis.

[71] Zhan X. (2015). Evaluation of Building Life Cost and Fuzzy Comprehensive Evaluation of Earthquake Direct Loss. Ph.D. Thesis.

[72] GB/T18208.4-2011 (2011). Post-Earthquake Field Works-Part 4: Assessment of Direct Seismological Loss.

[73] Ma Y., Zhao G. (2008). Risk Analysis and Management of Earthquake Disaster.

